# Cutaneous *Tsukamurella tyrosinosolvens* infection in an immunocompetent patient

**DOI:** 10.1016/j.jdcr.2024.04.027

**Published:** 2024-04-26

**Authors:** Shaliz Aflatooni, Alison H. Kucharik, Kayla M. Fourzali, Leslie Turner, Catherine Kowalewski

**Affiliations:** a University of South Florida Morsani College of Medicine, Tampa, Florida; b Department of Dermatology and Cutaneous Surgery, University of South Florida Morsani College of Medicine, Tampa, Florida; c Department of Dermatopathology, James A. Haley Veterans’ Hospital, Tampa, Florida; d Department of Dermatology, James A. Haley Veterans’ Hospital, Tampa, Florida

**Keywords:** cutaneous infection, minocycline, *T. tyrosinosolvens*, *Tsukamurella*

## Introduction

*Tsukamurellatyrosinosolvens* is a Gram-positive, weakly acid-fast bacillus belonging to the class Actinomycetes. Species within the *Tsukamurella* genus are environmental saprophytic bacteria found in soil and water. In the context of human infections, *Tsukamurella* has been recognized as an opportunistic pathogen, particularly affecting individuals with immunosuppression or indwelling medical devices.^1^^,^^2^ Further, the literature predominantly reports *Tsukamurella* infections in the context of bacteremia, respiratory tract infections, and catheter-related bloodstream infections.^3^ Consequently, cutaneous infections are uncommon and have only been documented in cases involving the species *Tsukamurella paurometabolum*.^4^ To date, there are no reported instances of cutaneous infections caused by *T tyrosinosolvens*. In this report, we present a 49-year-old immunocompetent woman with a nonhealing cutaneous wound as a result of *T tyrosinosolvens* infection.

## Case presentation

A 49-year-old immunocompetent woman presented to her primary care provider for a nonhealing lesion on her right hand due to a presumed spider bite. Review of systems was normal and vital signs were within normal limits. The lesion initially improved with doxycycline, however, subsequently worsened. She sought emergent care, where she was diagnosed with an abscess and prescribed a 10-day course of cephalexin and sulfamethoxazole-trimethoprim without improvement.

**Fig 1:**
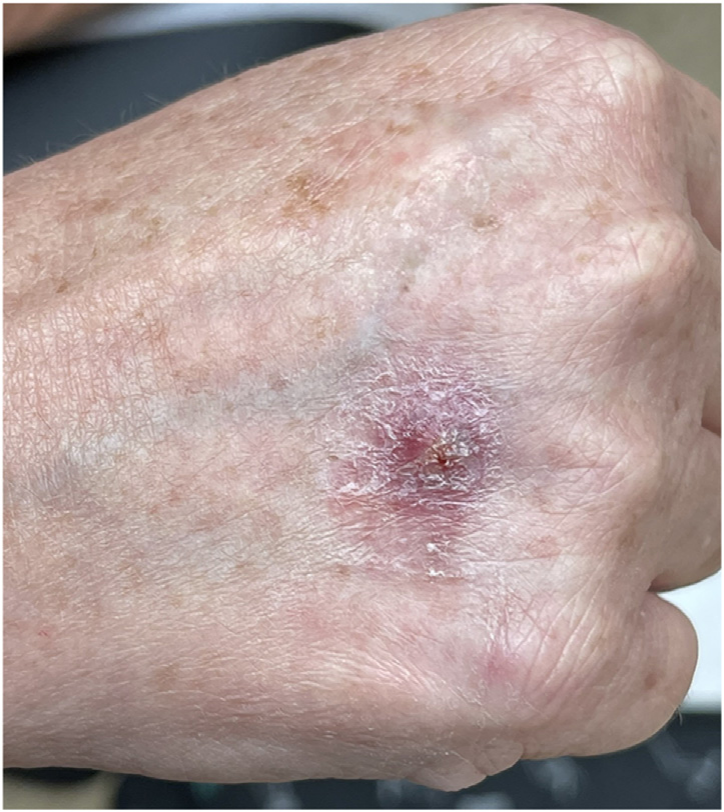
Erythematous-to-violaceous papule with overlying hemorrhagic crust and scale on dorsal aspect of the right hand lesion with culture proven *Tsukamurella tyrosinosolvens*.

**Fig 2:**
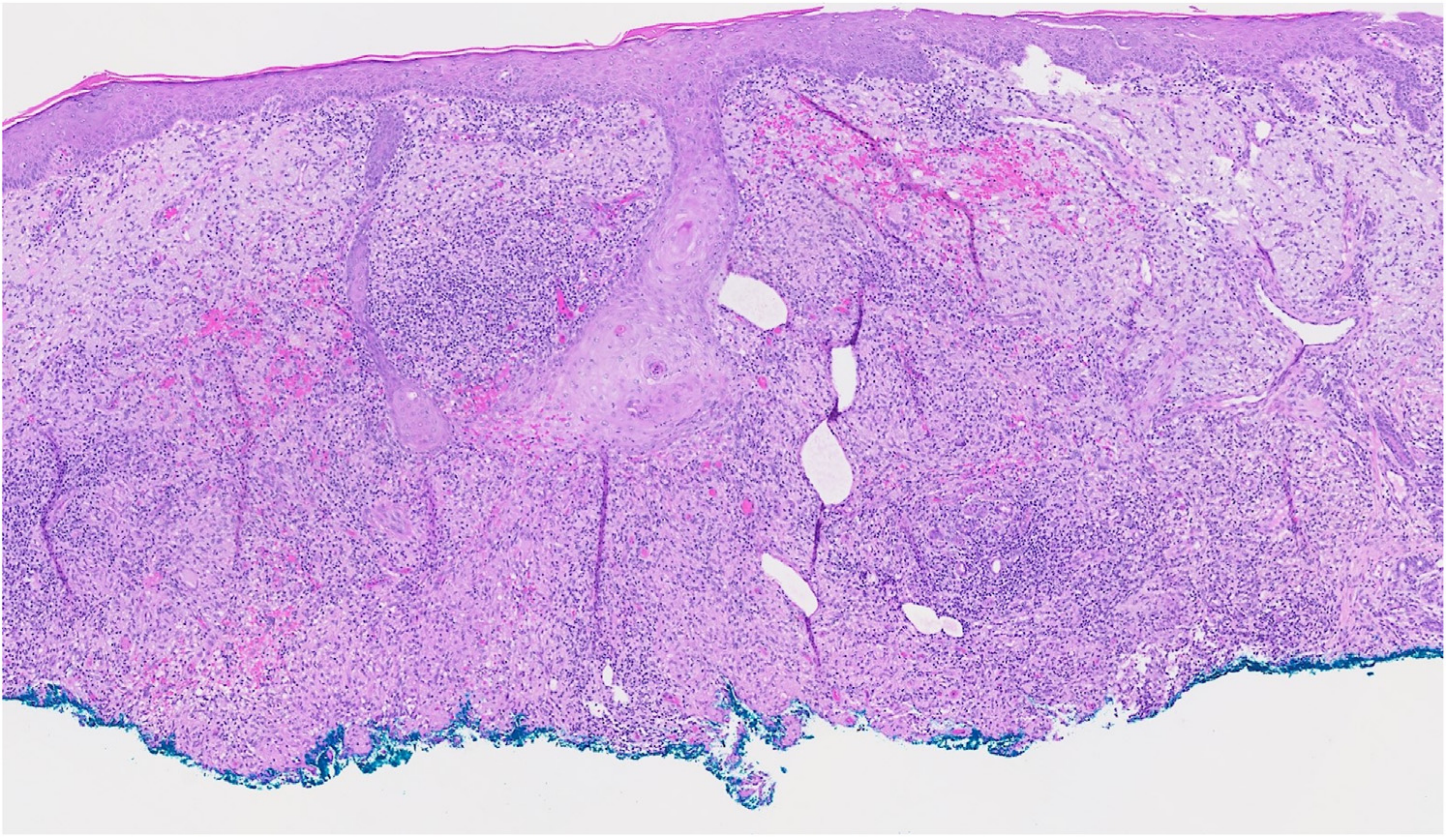
Pseudoepitheliomatous hyperplasia with an associated dense mixed inflammatory infiltrate (original magnification: ×5).

**Fig 3:**
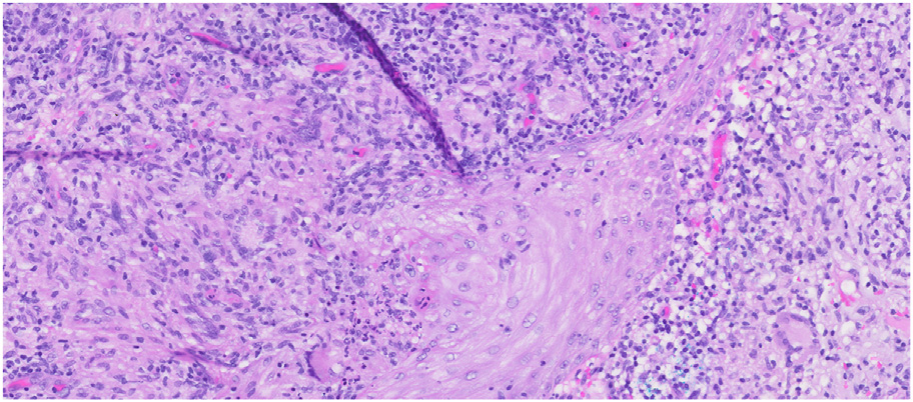
Numerous multinucleated giant cells are seen within the mixed dermal inflammation (original magnification: ×20).

Two months later, she presented to the dermatology clinic with a 9 mm erythematous-to-violaceous eroded papule with overlying hemorrhagic crust and scale on the dorsal aspect of the right hand (Fig 1). Initial differential diagnosis included cellulitis or abscess due to bacterial infection, deep fungal infections, and cutaneous malignancy, such as squamous cell carcinoma. Social history revealed that she owns tropical fish and cleans aquariums, raising concern for *Mycobacterium marinum* or another aquatic-associated organism. A shave biopsy for histopathologic analysis was performed, revealing a reactive atypical squamous proliferation and a prominent granulomatous and suppurative infiltrate highly suggestive of an infectious process (Fig 2, Fig 3 and Fig 2, Fig 3). Fungal and bacterial stains, including staining for acid-fast bacilli, were negative for organisms on histopathology. Empiric oral minocycline 100 mg twice daily was initiated at that time due to concern of *M marinum* infection.

**Fig 4:**
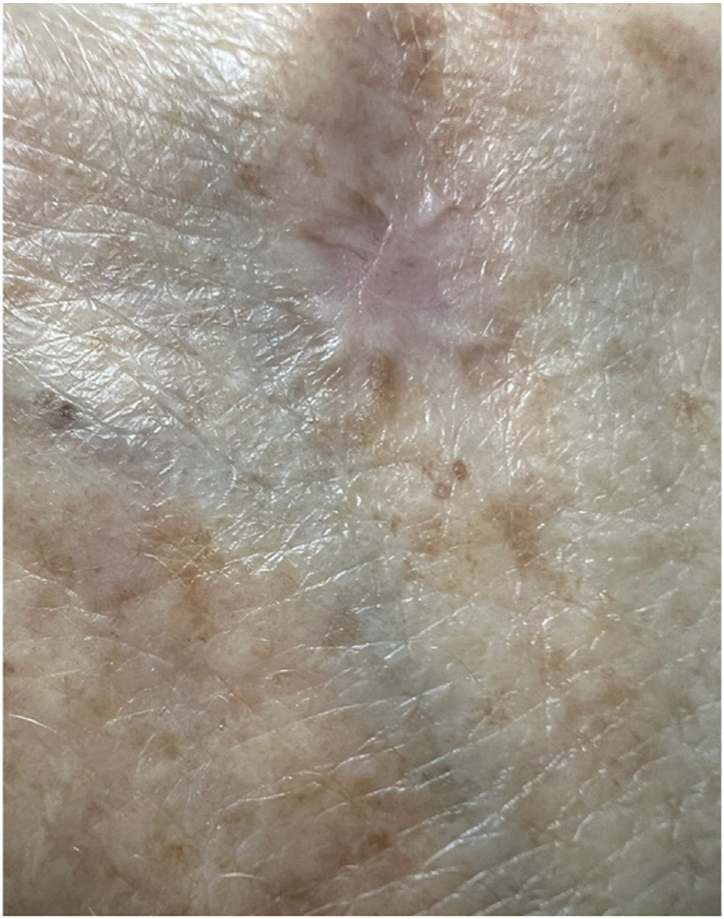
Reepithelialization of dorsal aspect of the right hand 5.5 months after starting minocycline.

Infectious tissue workup included bacterial and mycobacterial tissue culture, which resulted in positivity for acid-fast bacilli and ultimately showed growth of *T tyrosinosolvens*. Given the rarity of the pathogen, Infectious Disease was consulted, who agreed with extension of her minocycline course to 5.5 months. At her most recent follow-up, examination revealed complete reepithelialization of the lesion (Fig 4).

## Discussion

Human infection with *T tyrosinosolvens* is exceptionally rare. In documented cases in the literature, this bacterium has been isolated primarily in immunocompromised individuals with conditions such as gastric cancer, chronic pulmonary infections, or in those carrying cardiac implants or intravascular catheters.^5^, ^6^, ^7^ This report contributes a unique case of *T tyrosinosolvens*, notably in an immunocompetent patient with infection limited to cutaneous involvement.

*Tsukamurella* species are saprophytic in nature, meaning they feed on decaying matter and thrive in soil and water environments.^8^ *Tsukamurella* has been previously isolated from the sea, so it is plausible that our patient acquired this environmental saprophyte during the maintenance of her tropical fish water tank.^9^

*Tsukamurella* shares common features with other mycolic acid containing organisms with in the Actinomycetes class such as *Nocardia, Gordonia, Rhodococcus, Corynebacterium*, and *Mycobacterium*, making identification challenging.^10^ *T tyrosinosolvens* is considered a weakly acid-fast bacillus. In our case, the initial histopathology staining for acid-fast bacilli was unrevealing; however, more sensitive culture for acid-fast bacilli was positive. The special stains only look at a few microns of the tissue, making it highly variable, especially in the setting of an infectious process with very few organisms.

There is an absence of standardized guidelines for treating *Tsukamurella* infections due to the scarcity of cases and paucity of information on the susceptibility of this bacteria to antimicrobial agents. Recently, Yu et al^10^ demonstrated the potent *in vitro* activity of quinolones, trimethoprim/sulfamethoxazole, amikacin, minocycline, linezolid, and tigecycline against *Tsukamurella*. In our case, the patient underwent a prescribed 5.5-month minocycline course, leading full clinical resolution and complete reepithelialization of the wound. This outcome provides additional evidence supporting minocycline as a promising treatment approach for *T tyrosinosolvens* and offers valuable guidance for future cases.

## Conflicts of interest

None disclosed.

## References

[1] Ochi F., Tauchi H., Moritani K., Miyamoto H., Ohkusu K., Ishii E. (2015). *Tsukamurella inchonensis* infection in a child with Hodgkin’s lymphoma. Pediatr Int.

[2] Alcaide M.L., Espinoza L., Abbo L. (2004). Cavitary pneumonia secondary to *Tsukamurella* in an AIDS patient. First case and a review of the literature. J Infect.

[3] Safaei S., Fatahi-Bafghi M., Pouresmaeil O. (2018). Role of *Tsukamurella* species in human infections: first literature review. New Microbes New Infect.

[4] Granel F., Lozniewski A., Barbaud A. (1996). Cutaneous infection caused by *Tsukamurella paurometabolum*. Clin Infect Dis.

[5] Matsumoto T., Shiraishi M., Yoshimura H. (2006). *Tsukamurella tyrosinosolvens* cultured from sputum of a patient who received total gastrectomy for gastric cancer. Kekkaku.

[6] Yassin A.F., Rainey F.A., Burghardt J. (1997). *Tsukamurella tyrosinosolvens* sp. nov. Int J Syst Bacteriol.

[7] Sheridan E.A., Warwick S., Chan A., Dall’Antonia M., Koliou M., Sefton A. (2003). *Tsukamurella tyrosinosolvens* intravascular catheter infection identified using 16S ribosomal DNA sequencing. Clin Infect Dis.

[8] Goodfellow M., Kumar Y. (2012).

[9] Olson J.B., Harmody D.K., Bej A.K., McCarthy P.J. (2007). *Tsukamurella spongiae* sp. nov., a novel actinomycete isolated from a deep-water marine sponge. Int J Syst Evol Microbiol.

[10] Yu S., Ding X., Hua K. (2023). Systematic investigation of the emerging pathogen of *Tsukamurella* species in a Chinese tertiary teaching hospital. Microbiol Spectr.

